# Psychiatrische Konsiliar- und Liaisontätigkeit in der Notaufnahme/Rettungsstelle

**DOI:** 10.1007/s00115-025-01823-9

**Published:** 2025-04-07

**Authors:** Kathrin Nickel, Katharina Domschke, Dieter Ebert

**Affiliations:** 1 Klinik für Psychiatrie und Psychotherapie, Universitätsklinikum Freiburg, Medizinische Fakultät, Albert-Ludwigs-Universität Freiburg, Freiburg, Deutschland; 2 Deutsches Zentrum für Psychische Gesundheit (DZPG), Partnerstandort Berlin/Potsdam, Berlin, Deutschland

**Keywords:** Psychische Erkrankung, Psychiatrischer Konsildienst, SMART Medical Clearance, Psychiatrischer Notfall, Standardisierung, Psychiatric disorders, Psychiatric consultation service, SMART medical clearance, Standardization, Psychiatric emergency

## Abstract

In somatischen Notaufnahmen ist die Prävalenz psychiatrischer Notfälle mit 5–10 % hoch. Der psychiatrische Konsiliar- und Liaisondienst erfüllt in somatischen Notaufnahmen eine zentrale Funktion in der interdisziplinären Versorgung von Notfallpatienten mit primär psychischen Erkrankungen, organisch bedingten psychischen Störungen sowie somatischen Erkrankungen mit psychischer Komorbidität. Während die Konsiliartätigkeit die Beratung anderer Fachdisziplinen umfasst, ermöglichen Konsiliar- und Liaisondienste eine kontinuierliche Mitbehandlung von Patienten. Angepasst an die Organisation als Konsiliar- oder Konsiliar- und Liaisondienst kann eine effiziente, zielgerichtete, sichere und ökonomische Abklärung psychischer Symptome bzw. ein Screening möglichst vieler Patienten mit psychischen Symptomen erfolgen, um einen Zugang zu einer psychiatrischen Behandlung zu ermöglichen. Der psychiatrische Konsiliar- und Liaisondienst kann auch bei der Entscheidung über notwendige somatische Untersuchungen vor einer psychiatrischen Weiterbehandlung unterstützen. Das „SMART Medical Clearance Formular“ stellt einen Ansatz zur Standardisierung somatischer Abklärungen bei Patienten mit psychischen Symptomen in Notaufnahmen dar. Bezüglich der psychiatrischen Beurteilung von Patienten in somatischen Notaufnahmen bedarf es einer weiteren Standardisierung empfohlener somatischer Abklärungen unter Berücksichtigung individueller Faktoren sowie der Klärung, ob somatische Symptome eventuell eine psychiatrische Erkrankung als zugrunde liegende Ursache haben. Zudem sollten Richtlinien festlegen, welche Patienten in Notaufnahmen eine konsiliarische Beurteilung erhalten sollten.

## Abstract (en)

In somatic emergency departments, the prevalence of psychiatric emergencies is high, ranging from 5–10%. The psychiatric consultation and liaison service plays a central role in the interdisciplinary care of emergency patients in somatic emergency departments. It addresses patients primarily with psychiatric disorders, organically caused psychiatric conditions and somatic illnesses with psychiatric comorbidities. While consultation activities involve advising other medical disciplines, consultation and liaison services enable continuous treatment of patients. Depending on the organization as a consultation or combined consultation and liaison service, the aim is to ensure efficient, targeted, safe and cost-effective evaluation of psychiatric symptoms and/or to screen as many patients as possible with psychiatric symptoms, facilitating access to psychiatric treatment. Psychiatric consultation and liaison services can furthermore support decisions regarding necessary somatic evaluations prior to psychiatric treatment. The “SMART medical clearance form” provides a standardized approach to somatic evaluations for patients with psychiatric symptoms in emergency departments. To improve psychiatric assessments of patients in somatic emergency departments, further standardization of the recommended somatic examinations and the evaluation of whether somatic symptoms can be associated with an underlying psychiatric disorder are needed. In addition, guidelines should establish which patients require a psychiatric consultation assessment in emergency departments.

In Notaufnahmen spielt der psychiatrische Konsiliar- und Liaisondienst eine zentrale Rolle in der integrierten Versorgung von Patienten mit primären psychischen Erkrankungen, organisch bedingten psychischen Störungen sowie somatischen Erkrankungen mit psychischer Komorbidität. Protokolle wie das „SMART Medical Clearance Formular“ stellen einen Ansatz dar, um durchzuführende somatische Abklärungen von Patienten mit psychischen Symptomen in Notaufnahmen zu standardisieren. Eine strukturierte interdisziplinäre Kooperation fördert eine frühzeitige Diagnostik, optimierte Therapie und effiziente Versorgung von Patienten mit somatischer und psychischer Komorbidität.

## Psychiatrische Konsiliar- und Liaisontätigkeit in der Notaufnahme

Psychische Erkrankungen sind in Notaufnahmen sowohl als primäre Erkrankung als auch als Begleiterscheinungen somatischer Erkrankungen präsent. Eine weitere Herausforderung in Notaufnahmen stellen Patienten dar, welche körperliche Symptome zeigen, für die kein organisches Korrelat gefunden wird und denen eine psychische Erkrankung zugrunde liegt [7]. Psychische Symptome oder eigenständige psychische Erkrankungen als Komorbiditäten somatischer Erkrankungen können die medizinische Versorgung erschweren, indem sie mit längeren Krankenhausaufenthalten, erhöhten Wiederaufnahmeraten und höheren medizinischen Kosten assoziiert sind [6].

Die psychiatrische Konsiliar- und Liaisontätigkeit in der Notaufnahme ermöglicht eine interdisziplinäre Zusammenarbeit zwischen verschiedenen medizinischen Fachdisziplinen, um eine umfassende multimodale Versorgung der Patienten insbesondere unter Berücksichtigung der häufig bestehenden psychischen und somatischen Komorbiditäten sicherzustellen. Der psychiatrische Konsiliar- und Liaisondienst in somatischen Notaufnahmen stellt häufig den initialen psychiatrischen Kontakt dar und kann somit einen Zugang zu einer frühzeitigen psychiatrischen Diagnostik und Therapie ermöglichen.

Während die Konsiliartätigkeit eine Beratung anderer medizinischer Fachdisziplinen beinhaltet, umfasst die Liaisontätigkeit eine interdisziplinäre kontinuierliche Zusammenarbeit und Mitbehandlung der Patienten in somatischen Notaufnahmen.

Von der Organisationsstruktur als psychiatrischer Konsiliar- bzw. Konsiliar- und Liaisondienst an einer Klinik sind die Vorgehensweisen in somatischen Notaufnahmen abhängig, da Konsiliardienste teilweise andere Aufgaben als Liaisondienste erfüllen. Hierbei stellt sich in der Praxis bei psychiatrischen Konsiliardiensten die Frage, wie eine Beratung anderer medizinischer Fachdisziplinen auch unter Berücksichtigung ökonomischer Faktoren effizient und sicher organisiert werden kann und welche Patienten in somatischen Notaufnahmen psychiatrisch beurteilt werden sollten. Gleichzeitig kann ein Konsiliar- und Liaisondienst ein Screening ermöglichen, um möglichst viele Patienten mit psychischen Störungen zu identifizieren und ihnen eine weiterführende Behandlung zukommen zu lassen.

## Prävalenz psychischer Erkrankungen in somatischen Notaufnahmen

Die Prävalenz psychischer Erkrankungen bei Patienten, welche sich im Allgemeinkrankenhaus in stationärer Behandlung befinden, wurde in einem systematischen „Umbrella-Review“ auf ca. ein Drittel geschätzt [9]. Dabei lag die Prävalenz depressiver Störungen (Major Depression) bei 12–20 % [9], während die Prävalenz irgendeiner Angststörung auf 8 %, einer generalisierten Angststörung auf 5 %, einer Panikstörung auf 3 % [9, 13] sowie einer Demenz zwischen 3 % und 63 % geschätzt wurde [9]. Depressionen zählen zu den häufigsten Komorbiditäten zahlreicher chronischer somatischer Erkrankungen, darunter Tumorerkrankungen sowie kardiovaskuläre, metabolische, inflammatorische und neurologische Störungen. In diesen Patientenpopulationen übersteigt die Prävalenz von Depressionen jene in der Allgemeinbevölkerung [5]. Dies hebt die zentrale Bedeutung des psychiatrischen Konsiliar- und Liaisondienstes für die Tätigkeit in somatischen Notaufnahmen hervor.

Der Anteil psychiatrischer Notfälle in somatischen Notaufnahmen wird auf ca. 5–10 % geschätzt [7, 8].

Psychiatrische Notfälle nehmen aufgrund einer allgemeinen Zunahme der Inanspruchnahme von Notaufnahmen, einer Abnahme stützender psychosozialer Faktoren und einem veränderten Angebot in der ambulanten Patientenversorgung kontinuierlich zu [11].

Eine retrospektive Analyse psychiatrischer Notfälle in interdisziplinären Notaufnahmen zeigte, dass ca. 30 % der Patienten, die in der S2k-Leitlinie Notfallpsychiatrie (2019) aufgeführten Kriterien für „absolute oder relative Notfallindikationen“ nicht erfüllten [7]. Von den nicht die Notfallkriterien erfüllenden Patienten stellten sich nur ca. 10 % zuvor bei ihrem Hausarzt vor, und in ca. 88 % der Fälle erfolgte eine Selbstvorstellung in der Notaufnahme [7]. Der große Anteil an Patienten in Notaufnahmen, welche die Kriterien für „absolute oder relative Notfallindikationen“ nicht erfüllen, weist darauf hin, dass ein mögliches Defizit im Bereich der Steuerung und Information im Notfallsystem besteht [7].

## Interdisziplinäre Kooperation – somatische Notaufnahme und psychiatrischer Konsiliar- und Liaisondienst

Der Ablauf der Kooperation zwischen somatischen Notaufnahmen und dem psychiatrischen Konsiliar- und Liaisondienst kann durch zwei Faktoren beeinträchtigt werden:

- Fall 1: Ein Patient mit einer primär behandlungsbedürftigen somatischen Erkrankung wird nicht hinreichend organisch „abgeklärt“ einer psychiatrischen Behandlung zugewiesen.
- Fall 2: Ein Patient mit einer akut behandlungsbedürftigen psychischen Erkrankung wird ohne weiterführende psychiatrische Beurteilung in einer somatischen Klinik aufgenommen.

### Fall 1: Somatische Erkrankung wird „übersehen“ und der Patient in eine psychiatrische Klinik eingewiesen

#### Beispiel.

Ein Patient wird mit Verwirrtheit bei vorbekannter paranoider Schizophrenie in der Notaufnahme vorgestellt. Es wird eine sofortige Übernahme in eine psychiatrische Klinik initiiert. Das Aufnahmelabor zeigt dort eine ausgeprägte intensivmedizinisch behandlungspflichtige Hyponatriämie.

#### Somatische Abklärung von Patienten mit psychischen Symptomen

Zusätzlich zur Schwierigkeit der Triage psychiatrischer Notfälle existieren bisher keine klaren Richtlinien, welche somatischen Untersuchungen in Notaufnahmen bei Patienten mit psychischen Symptomen und Erkrankungen durchgeführt werden sollten. Die diesbezüglich nichtstandardisierten Vorgehensweisen führen häufig zu Verzögerungen in der Patientenversorgung [12]. Hierbei kann der Konsiliar- und Liaisonpsychiater in der somatischen Notaufnahme im Rahmen einer effektiven interdisziplinären Kooperation unterstützend und beratend tätig sein. Insbesondere ist es essenziell, dass vor einer Zuweisung in eine psychiatrische Klinik potenzielle somatische Ursachen für den aktuellen psychischen Zustand ausgeschlossen bzw. gegebenenfalls entsprechend behandelt worden sind.

##### Das „SMART Medical Clearance Formular“.

Zur Optimierung der Zusammenarbeit zwischen somatischen und psychiatrischen Fachdisziplinen und um Verzögerungen in der Versorgung von Patienten mit psychischen Erkrankungen in somatischen Notaufnahmen zu vermeiden, wurde das sogenannte „SMART Medical Clearance Formular“ von der Sierra Sacramento Valley Medical Society in Kooperation mit Notärzten und Psychiatern in den USA entwickelt.

Beim „SMART Medical Clearance Formular“ handelt es sich um ein auf evidenzbasierten Studien entwickeltes Protokoll, um die somatische Abklärung von Patienten, die sich mit primär psychischen Beschwerden in Notaufnahmen vorstellen, zu standardisieren. Ziel ist es, nicht notwendige somatische Untersuchungen zu vermeiden und gleichzeitig die Wartezeiten in Notaufnahmen für alle Patienten zu verkürzen [15]. Es berücksichtigt daher die folgenden Kategorien:

- Verdacht auf das Vorliegen einer neu aufgetretenen psychischen Erkrankung (*S* – „suspect new onset psychiatric condition“),
- medizinische Symptomatik, welche ein weiteres Screening erfordert (*M* – „medical conditions that require screening“),
- pathologische Werte (*A* – „abnormal“),
- eine „Risikokonstellation“ (*R* – „risky presentation“) sowie
- die Erforderlichkeit des Erhebens von Medikamentenspiegeln im Blut (*T* – „therapeutic levels needed“).

**Abb. 1:**
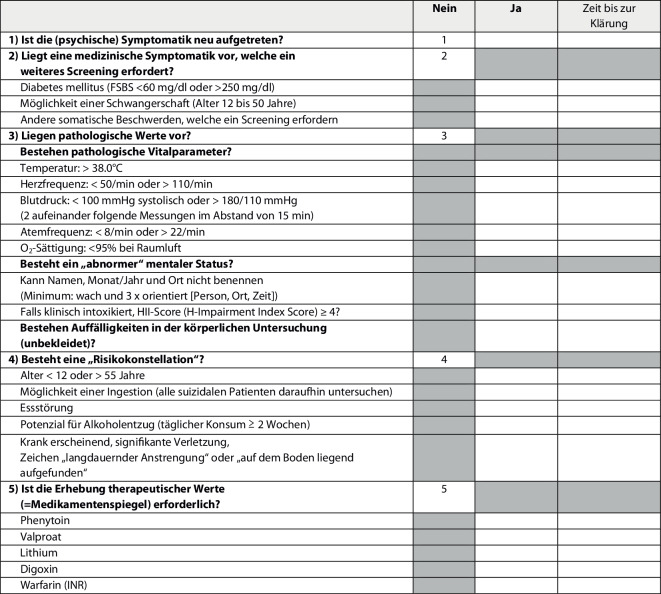
„SMART Medical Clearance Formular“ – erhobene Parameter [15]. Wenn alle fünf SMART-Kategorien mit „NEIN“ bewertet werden (nSMART), gilt der Patient als „medizinisch abgeklärt“ und es sind keine weiteren Tests erforderlich. Wenn eine Kategorie mit „JA“ angekreuzt wird (pSMART), müssen entsprechende Untersuchungen durchgeführt und/oder eine Begründung in der Patientenakte vermerkt und die Zeit bis zur Klärung dokumentiert werden. Vor einer psychiatrischen Weiterbehandlung ist eine weiterführende somatische Abklärung indiziert. *FSBS* „Finger Stick Blood Sugar“, *INR* International Normalized Ratio. Übersetzung durch die Autoren

Die im „SMART Medical Clearance Formular“ erfassten Parameter sind in Abb. 1 zusammengefasst. Sollte kein SMART-Kriterium zutreffen, gilt der Patient als „medizinisch abgeklärt“ und es sind keine weiteren Untersuchungen vor Weiterleitung in eine psychiatrische Behandlung erforderlich [2, 14, 15].

Studien zeigen, dass die Aufenthaltsdauer in Notaufnahmen durch Zuhilfenahme des „SMART Medical Clearance Formulars“ um ca. 15 % verkürzt werden konnte [2].

Eine aktuelle Studie des Luzerner Kantonsspitals in der Schweiz evaluierte das „SMART Medical Clearance Formular“ und stellte fest, dass es ein vielversprechendes Instrument darstellt, um eine sichere und standardisierte Weiterleitung und Behandlung von Patienten mit psychischen Symptomen in somatischen Notaufnahmen zu ermöglichen [4]. Während bei 134 von 674 in die Studie eingeschlossenen Patienten, welche sich mit psychischen Beschwerden im Kantonsspital Luzern vorstellten, kein SMART-Parameter zutraf (nSMART), erfüllten 540 mindestens einen Parameter im „SMART Medical Clearance Formular“ (pSMART). Hierbei wurden am häufigsten pathologisch veränderte Vitalparameter bzw. Auffälligkeiten im körperlichen/mentalen Untersuchungsbefund sowie „kritische Risikokonstellationen“ festgestellt. Etwa 4 % der sich in der Notaufnahme vorstellenden Patienten zeigte psychische Symptome als Vorstellungsgrund. Diese wurden mit dem „Canadian Emergency Department Information System“ (CEDIS), welches standardisiert Vorstellungsgründe in einer Notaufnahme erfasst, erhoben. Hierbei stellten „Depressivität/Suizidalität/Selbstschädigung“ (ca. 31 %) und „sonderbares Verhalten“ (ca. 19 %) die häufigsten Symptompräsentationen dar, wobei in ca. 28 % der Fälle die Symptomatik erstmalig aufgetreten war.

Während in der pSMART-Gruppe innerhalb von 30 Tagen 4 Todesfälle und 90 Hospitalisationen aus medizinischen Gründen registriert wurden, traten in der nSMART-Gruppe keine Todesfälle oder Hospitalisationen aus medizinischen Gründen auf. Die Verweildauer in der Notaufnahme betrug bei der nSMART-Gruppe im Median 181 min, in der pSMART-Gruppe 240 min. Bei den 674 Patienten der pSMART-Gruppe wurden bei 435 Patienten labormedizinische Abklärungen, bei 285 12-Kanal-Elektrokardiogramme (EKGs) und bei 108 eine bildgebende Diagnostik initiiert, wobei sich bei ca. 10 % pathologisch relevante Laborwerte, bei ca. 11 % ein pathologisches EKG und bei ca. 4 % relevante Auffälligkeiten in der Bildgebung zeigten. Das „SMART Medical Clearance Formular“ wird als wertvolles Instrument eingestuft, um die somatischen Untersuchungen in Notaufnahmen bei Patienten mit initialen psychiatrischen Vorstellungsgründen zu standardisieren und optimieren [4].

##### Weitere Empfehlungen für die psychiatrisch-somatische Kooperation in Notaufnahmen.

Die Wahrscheinlichkeit, somatische Erkrankungen zu übersehen, besteht am ehesten bei psychiatrischen Notfällen. Hierbei ist ein psychiatrischer Notfall als medizinische Situation definiert, bei der eine unmittelbare Gefahr für das eigene Leben oder die Gesundheit oder die ihrer Umgebung bei plötzlichem Auftreten oder Verschlechterung einer psychischen Störung besteht, welche einer unverzüglichen Diagnostik und/oder Behandlung bedarf [10].

Nach den in der S2k-Leitlinie Notfallpsychiatrie [3] aufgeführten „Ausführungen der Bundesärztekammer“ [1] werden ein erfolgter Suizidversuch, konkrete Suizidpläne oder -vorbereitungen, ein hochgradiger Erregungszustand, Aggressivität/Gewalttätigkeit im Rahmen psychischer Erkrankungen, schwere Intoxikationen und das Delir als „absolute Notfallindikationen“ eingeordnet. Währenddessen werden Verwirrtheit, Entzugssyndrome ohne Delir, Suizidgedanken ohne konkrete Pläne, Angst und Panikstörung sowie eine akute Belastungsreaktion den „relativen Notfallindikationen“ zugeordnet (S2k-Leitlinie Notfallpsychiatrie [3]). Nach Pajonk und Messer [10] werden Suizidalität, Erregungszustände, Delir, Bewusstseinsstörungen und Verwirrtheit, Intoxikationen und andere substanzbedingte Störungen, paranoid-halluzinatorische Syndrome, manische Syndrome, Stupor und Katatonie, Angststörungen, psychosoziale Krisen und Traumatisierung sowie die Anorexie als typische notfallpsychiatrisch relevante Störungen und Syndrome eingeordnet.

Im Rahmen einer systematischen Literaturübersicht wurden zusätzlich zur Anwendung standardisierter Algorithmen (z. B. „SMART-Formular“) zur Erhebung somatischer Beschwerden bei Patienten mit psychischen Symptomen in Notaufnahmen weitere Vorschläge zur somatischen Beurteilung von Patienten vor potenzieller Weiterleitung in eine psychiatrische Behandlung herausgearbeitet. Diese Empfehlungen werden vom Wisconsin Chapter of the American College of Emergency Physicians und der Wisconsin Psychiatric Association befürwortet.

Sie umfassen die folgenden Aspekte:

1. Eine ausführliche Anamnese und eine körperliche Untersuchung sollten die Mindestanforderungen für die meisten medizinischen Beurteilungen darstellen.
2. Entscheidungen bezüglich weiterer diagnostischer Abklärungen sollten durch klinische Informationen geleitet sein, und es sollten keine pauschalen Anforderungen für routinemäßige Tests erfolgen.
3. In Notaufnahmen tätige Ärzte sollen sich der „begrenzten“ medizinischen Möglichkeiten psychiatrischer Kliniken bewusst sein. Die Durchführung adäquater diagnostischer Tests, welche in diesen Kliniken nicht verfügbar sind, kann sinnvoll sein, es sollte jedoch zu keiner Verzögerung im Patiententransfer kommen.
4. Es sollte ein regelmäßiger interdisziplinärer direkter Austausch zwischen in Notaufnahmen tätigen Ärzten sowie Psychiatern erfolgen [12].

### Fall 2: Psychische Erkrankung wird „übersehen“ und der Patient keiner psychiatrischen Behandlung zugewiesen

#### Beispiel.

Ein Patient wird aufgrund ausgeprägter Bauchschmerzen in der Notaufnahme vorgestellt. Alle Untersuchungen inklusive u. a. einer Laboruntersuchung, Sonographie und Koloskopie ergeben keine auffälligen Befunde. Der hinzugezogene Konsiliarpsychiater exploriert die Symptome einer schweren depressiven Episode mit Zönästhesien.

Bei Patienten, bei welchen nach vollständiger somatischer Abklärung keine körperliche Ursache für die vorliegende Symptomatik gefunden werden konnte, sollte die Möglichkeit einer zugrunde liegenden psychischen Erkrankung in Betracht gezogen werden, um diese nicht zu übersehen und gegebenenfalls eine weiterführende psychiatrische Behandlung zu initiieren.

#### Psychiatrische Beurteilung von Patienten in somatischen Notaufnahmen im Konsiliar- und Liaisondienst

Bei der Beurteilung von Patienten in der somatischen Notaufnahme durch den psychiatrischen Konsiliar- und Liaisondienst ist es entscheidend, festzulegen, bei welchen Zuständen es sich um psychiatrische Notfälle handelt und bei welchen Vorstellungen eine konsiliarpsychiatrische Beurteilung indiziert ist. Es muss evaluiert werden, bei welchen Vorstellungen von Patienten mit psychischen Beschwerden eine unverzügliche Abklärung somatisch bzw. psychiatrisch erfolgen muss und in welchen Fällen eine elektive ambulante psychiatrische Vorstellung und Weiterbehandlung erfolgen kann.

Bisher liegen keine standardisierten Empfehlungen vor, bei welchen Vorstellungen von Patienten mit primär psychischen Beschwerden in somatischen Notaufnahmen eine Beurteilung durch den psychiatrischen Konsiliar- und Liaisondienst erfolgen muss, sollte bzw. kann.

Sind die Kriterien einer akuten Eigen- oder Fremdgefährdung im Rahmen einer psychischen Störung erfüllt, ist eine konsiliarische psychiatrische Beurteilung in jedem Fall indiziert.

##### Psychiatrische konsiliarische Beurteilung in somatischen Notaufnahmen.

Bei der Durchführung konsiliarpsychiatrischer Beurteilungen in somatischen Notaufnahmen sollten die folgenden Parameter erhoben und berücksichtigt werden:

- aktuelle Eigenanamnese ggf. ergänzt durch eine Fremdanamnese
- vollständiger psychopathologischer Befund mit diagnostischer Einordnung der vorliegenden Symptomatik
- Substanzanamnese
- psychiatrische Vorgeschichte
- aktuelle psychiatrische und somatische Medikation
- Familienanamnese
- Sozialanamnese

Zudem sollte geprüft werden, welche somatischen Befunde (z. B. körperliche Untersuchung, Laboruntersuchung, Drogenscreening, EKG, EEG, zerebrale Bildgebung, Liquoruntersuchung) bereits vorliegen und ob eine weiterführende organische Abklärung von psychiatrischer Seite vor einer Verlegung in eine psychiatrische Klinik oder der Vermittlung in eine ambulante psychiatrische Behandlung empfohlen wird.

## Fazit für die Praxis

- Der psychiatrische Konsiliar- und Liaisondienst erfüllt eine essenzielle Funktion in der integrierten multidisziplinären Versorgung von Patienten in somatischen Notaufnahmen.
- Für die Zukunft bedarf es eines standardisierten Vorgehens bezüglich der somatischen Abklärung von Patienten in Notaufnahmen, die sich primär mit psychischen Beschwerden vorstellen.
- Das „SMART Medical Clearance Formular“ kann einen Beitrag zu einer effizienten somatischen Abklärung vor Zuweisung in eine psychiatrische Weiterbehandlung ermöglichen.
- Trotz Standardisierung müssen individuelle Faktoren bei der Entscheidung über indizierte somatische Abklärungen bei Patienten mit psychischen Beschwerden in Notaufnahmen berücksichtigt werden.
- Die Möglichkeit, dass somatischen Symptomen ohne nachweisbares organisches Korrelat eine psychische Erkrankung zugrunde liegen kann, muss berücksichtigt werden.

## Data Availability

Dieser Beitrag beinhaltet keine Studien an Menschen oder Tieren. Alle zugrunde liegenden Daten sind im Manuskript enthalten bzw. es wird auf die entsprechenden Quellen verwiesen.
